# The Nicotianamine Synthase Gene Is a Useful Candidate for Improving the Nutritional Qualities and Fe-Deficiency Tolerance of Various Crops

**DOI:** 10.3389/fpls.2018.00340

**Published:** 2018-03-27

**Authors:** Tomoko Nozoye

**Affiliations:** ^1^Center for Liberal Arts, Meiji Gakuin University, Kanagawa, Japan; ^2^Department of Global Agricultural Sciences, Graduate School of Agricultural and Life Sciences, The University of Tokyo, Tokyo, Japan

**Keywords:** calcareous soil, iron (Fe), zinc (Zn), Fe deficiency, nicotianamine (NA)

## Abstract

With the global population predicted to grow by at least 25% by the year 2050, the sustainable production of nutritious foods will be necessary for human health and the environment. Iron (Fe) is an essential nutrient for both plants and humans. Fe is poorly soluble, especially at high pH levels, at which it is difficult for living organisms to accumulate sufficient Fe. In plants, Fe deficiency leads to low yield and poor nutritional quality, as it significantly affects chlorophyll synthesis. Fe deficiency is a worldwide agricultural problem that is especially serious in soils with a high pH, such as calcareous soils, which comprise approximately 30% of cultivated soils worldwide. Genetic improvements in crops that can tolerate Fe deficiency will be required to meet the demands for crop production and could ultimately contribute to the amelioration of global warming. Nicotianamine (NA) is an Fe chelator in plants that is involved in metal translocation in the plant body. In mammals, NA inhibits angiotensin I-converting enzyme, which plays a key role in blood pressure control. It was recently shown that the enhancement of NA production using nicotianamine synthase is useful for increasing not only NA but also Fe and Zn levels in crops such as rice, soybean, and sweet potato. Additionally, these plants showed Fe-deficiency tolerance in calcareous soil. These results suggested that *NAS* overexpression simultaneously improves food quality and increases plant production. This review summarizes progress in generating crops overexpressing *NAS*.

## Increasing Fe Deficiency Tolerance Could Contribute to Food Security and Ameliorate Global Warming

Iron (Fe) is an essential nutrient for virtually all living organisms. Under aerobic conditions, Fe is oxidized to Fe(III) compounds, and their solubility in water is poor. Therefore, most Fe is not available to plants, although mineral soils contain 6% Fe by weight. Plants suffering Fe deficiency show leaf chlorosis, and their yield and nutritional quality are impaired dramatically (Marschner, 1995). This problem is exacerbated in soils with a high pH, such as calcareous soils, and is one of the major problems for crop production. Calcareous soils comprise approximately 30% of the cultivated soils worldwide (Chen and Barak, 1982). As the world population continues to increase, it is predicted that we will need 1.5 times more food in 2050 (High Level Expert Forum - How to Feed the World in 2050, 2009). It will be necessary to increase food production to meet this demand. However, the recent increase in atmospheric CO_2_ levels is causing climate change, and it will be difficult to expand the area occupied by cultivated land by removing forests, which already contribute 2% of the CO_2_ emissions (Intergovenmental Panel on Climate Change [IPCC], 2007). Problem soils, including calcareous soil, comprise 67% of the land globally (Food and Agriculture Organization of the United Nations). Improvements in plant growth in calcareous soils have great potential to increase the production of plant biomass and reduce atmospheric CO_2_ levels, which will ultimately contribute to ameliorating global warming (Conway, 2012; Schroeder et al., 2013). In addition, Fe is necessary for human health, and its deficiency causes anemia, an easily identified disease that is a serious health problem, especially in developing countries (Welch and Graham, 2004). Ultimately, Fe in the human diet comes from plant uptake from the soil. Therefore, biofortification, i.e., increasing the Fe level in food plants, would improve human health. Appropriate target levels of Fe might differ according to the crop and target countries, as food cultures differ. For a rice-based diet, the target concentration of Fe was estimated to be 14.5 µg/g dry weight (DW) in polished rice grains, which is more than twice the present amount in rice (6 µg/g DW) (Hotz and McClafferty, 2007; Johnson et al., 2011). Therefore, genetically modified crops that can tolerate Fe deficiency while taking up sufficient Fe from calcareous soils would have a great impact on food security and contribute to ameliorating global warming.

## Fe Acquisition Strategies in Plants

To acquire sparingly insoluble Fe from soil, plants have evolved two main strategies to acquire soil Fe (Marschner et al., 1986). Higher plants, not including graminaceous plants, which include soybean and sweet potato, are categorized as Strategy I plants, which reduce Fe(III) to Fe(II) by ferric-chelate reductases, and then take up Fe(II) via ferrous iron transporters IRT1 (Eide et al., 1996; Robinson et al., 1999; Vert et al., 2002). By contrast, graminaceous plants, including important staple crops such as rice, barley, and maize, are categorized as Strategy II plants, which produce and secrete Fe(III) chelators called mugineic acid family phytosiderophores (MAs) from their roots via the TOM1 transporter (Nozoye et al., 2011,Nozoye et al., 2013) and solubilize sparingly soluble Fe(III) in the rhizosphere (Takagi, 1976).

Nicotianamine (NA) is a non-proteinogenic amino acid that was first found in tobacco (Noma et al., 1971). NA chelates many metal cations, including Fe, zinc (Zn), copper (Cu), and manganese (Mn) (Beneš et al., 1983; Murakami et al., 1989; von Wirén et al., 1999). NA exists in all plants examined so far, including Strategy I and II plants (Hell and Stephan, 2003; Takahashi et al., 2003; Schuler et al., 2012), and plays an important role in the internal transport of metal nutrients (Mori et al., 1991; Kawai et al., 2001; Hell and Stephan, 2003; Takahashi et al., 2003; Suzuki et al., 2006; Schuler et al., 2012). In graminaceous plants, NA also serves as an intermediate for the biosynthesis of MAs (Takagi, 1976; Mori and Nishizawa, 1987; Shojima et al., 1990). NA synthase (NAS) converts three molecules of *S*-adenosyl methionine into NA (Shojima et al., 1989,Shojima et al., 1990; Higuchi et al., 1995). *NAS* genes were first isolated from barley and have subsequently been cloned from several plants species, including *Arabidopsis*, barley, rice, and maize (Herbik et al., 1999; Higuchi et al., 1999,Higuchi et al., 2001; Suzuki et al., 1999; Mizuno et al., 2003). Rice possesses three members: *OsNAS1-3*. *OsNAS1*, and *OsNAS2* are mainly expressed in Fe-deficient roots and shoots, whereas *OsNAS3* is also expressed in Fe-sufficient shoots (Inoue et al., 2003). It was suggested that all three have important roles in NA production under Fe-deficient conditions, although their roles might differ slightly.

## Nicotianamine Is Also an Attractive Functional Component in Human Health

In mammals, NA inhibits angiotensin I-converting enzyme (ACE), which plays a key role in blood pressure control (Kinoshita et al., 1993). ACE plays a role in the renin–angiotensin system in the maintenance of blood pressure and fluids, as well as electrolyte homeostasis (Re, 2004). ACE inhibitors are widely used as antihypertensive agents (Chirumamilla et al., 2001; Re, 2004) The oral administration of NA causes ACE inhibitory activity *in vitro* and antihypertensive effects in spontaneously hypertensive rats; moreover, the strength of ACE inhibition is correlated with NA content (Izawa et al., 2008). The inhibitory activity of NA against ACE is very strong (Kinoshita et al., 1993; Kataoka, 2005). Almost all vegetables contain more than 44 µg/g DW NA, which has the ability to inhibit ACE activity by more than 60–70% (Izawa and Aoyagi, 2012). In addition, NA from pumpkin not only improves hypertension, but also long-term memory function (Takada, 2011). In fact, brain-penetrating ACE inhibitors such as captopril reduce the incidence of Alzheimer’s disease in elderly hypertensive patients (Ohrui et al., 2004). Therefore, increased intake of NA through the diet could be effective for primary prophylaxis of hypertension and Alzheimer’s disease.

## Transgenic Approach to Increasing NA in Plants

Several reports have described transgenic plants generated by introducing the NAS gene (**Table 1**). The concentration of endogenous NA differs among crops (Izawa et al., 2008; Izawa and Aoyagi, 2012). The antihypertensive effect of NA was first identified in soybean (Kinoshita et al., 1993), which contains the highest amount of NA among the crops examined thus far (Izawa et al., 2008). In agreement with the endogenous NA level, the NA concentration in transgenic soybean was highest among the *HvNAS1*-overexpressing plants. The NA concentration in the transgenic soybean was increased to 768.1 µg/g DW in the seeds under the control of the cauliflower mosaic virus (CaMV) 35S promoter, which was four times higher than in non-transgenic (NT) seeds (Nozoye et al., 2014a). In sweet potato, overexpression of *HvNAS1* by the CaMV 35S promoter increased the NA concentration to 339.5 µg/g fresh weight (FW) in the leaves and 225.9 µg/g FW in the storage roots, which were 9.1 and 4.6 times higher, respectively, than in NT plants (Nozoye et al., 2017). In comparison, the NA concentration in *HvNAS1*-overexpressing tobacco (a dicot) by the CaMV 35S promoter was 78.9 µg/g FW in the leaves, which was 8.7 times higher than in NT plants (Kim et al., 2005). It was suggested that *NAS* genes are separated into two clusters between Gramineae and dicots and that the *NASs* in soybean and sweet potato were most similar in the dicot cluster (Nozoye et al., 2017). The *NASs* in soybean and sweet potato might have high enzymatic activity and produce more NA. The endogenous NA concentrations also differed among the tissues in rice (**Table 1**). The NA concentrations in leaves tended to be higher than that in the seeds. Consistent with the endogenous NA levels, the NA concentrations in leaves were also higher than those in seeds in *NAS*-overexpressing rice plants. Overexpression of *HvNAS1* by the CaMV 35S promoter increased the NA concentration to 75.8 µg/g DW in the polished seeds, which was 10.6 times higher than in NT seeds (Masuda et al., 2009). Overexpression of *HvNAS1* by the *OsActin1* promoter increased the NA concentration to 30.3 µg/g DW in the polished seeds, which were 16 and 5.1 times higher, respectively, than in NT plants (Masuda et al., 2009). By overexpressing rice *NAS* genes (*OsNAS1–3*) in rice under the control of an enhanced CaMV 35S promoter, the NA concentration in rice seeds increased to 210 µg/g DW, which was 9.3 times higher than in NT seeds (Johnson et al., 2011). In comparison, by overexpressing *OsNAS1* in rice under the control of the maize ubiquitin promoter, the NA concentration in rice leaves increased to 400 µg/g DW, which was 6.7 times higher than in NT leaves (Zheng et al., 2010). Using seed-specific expression of *OsNAS1* under the control of the rice glutelin promoter, the NA concentration in rice seeds increased to 65 µg/g DW, which was 5.2 times higher than in NT seeds (Zheng et al., 2010). This concentration was slightly lower than that in *NAS*-overexpressing rice seeds under the control of ubiquitous promoters. Additionally, in these plants, the NA concentration in shoots was not different from that in NT plants. These results suggest that it is possible to achieve a greater increase in NA in seeds by enhancing NA mobilization and translocation from leaves (and roots) to seeds. The average increase (fold change) in NA concentration was 7.6 and did not differ significantly among the crops, suggesting that the amount of endogenous NA is not a factor that limits the NA concentration. The combined enhancement of *NAS* and NA transporters could further elevate the NA level in the edible parts of the plant.

**Table 1:** NA, Fe, and Zn concentrations in NAS-overexpressing plants.

Promoter	Gene	Plant	Tissue	NA	Fe	Zn	Tolerance to Fe deficiency	Ref.
35S	HvNAS1	Soybean	Unpolished seeds	768.1 ± 82.9 (x4)	104 ± 9 (x2)	65 ± 3 (x1.5)	○	Nozoye et al., 2014a
35S	HvNAS1	Sweet potato	Leaves	339.5 ± 10.6^∗^ (x9.1)	52.9 ± 7 (x3)	16 ± 1.9 (x3)	○	Nozoye et al., 2017
35S	HvNAS1	Sweet potato	Storage roots	225.9^∗^ ± 140.2 (x4.6)	15.1 ± 4 (x2.1)	3.5 ± 0.7 (x3.5)	○	Nozoye et al., 2017
35S	HvNAS1	Tobacco	Leaves	78.9^∗^ ± 6 (x8.7)	3.7^∗^ ± 0.5 (x5)	9.6^∗^ ± 1.9 (x2.3)	nd	Kim et al., 2005
35S	HvNAS1	Rice	Polished seeds	75.8 ± 25.8 (x10.6)	9 ± 1.3 (x2)	45 ± 3.5 (x1.5)	nd	Masuda et al., 2009
OsActin1	HvNAS1	Rice	Leaves	24.3^∗^ ± 0.8 (x16)	170 ± 5 (x1)	25 ± 0.5 (x1)	nd	Masuda et al., 2009
OsActin1	HvNAS1	Rice	Polished seeds	30.3 ± 1.5 (x5.1)	6 ± 2.5 (x1)	37 ± 2.5 (x1)	nd	Masuda et al., 2009
35Sx2	OsNAS1	Rice	Unpolished seeds	96 to 115 (x6.4)	25 to 56 (x2.4)	40 to 59 (x1.9)	nd	Johnson et al., 2011
35Sx2	OsNAS2	Rice	Unpolished seeds	152 to 168 (x9.3)	19 to 81 (x3.5)	30 to 95 (x2.5)	nd	Johnson et al., 2011
35Sx2	OsNAS3	Rice	Unpolished seeds	174 to 210 (x11.7)	21 to 63 (x2.7)	30 to 79 (x2.1)	nd	Johnson et al., 2011
Maize ubiquitin promoter	OsNAS1	Rice	Leaves	400 ± 50 (x6.7)	28 ± 5 (x2.3)	120 ± 5 (x5.5)	nd	Zheng et al., 2010
Rice glutelin B1 promoter	OsNAS1	Rice	Unpolished seeds	41 to 65 (x5.2)	15.24 to 18.6 (x1.5)	31.74 to 36.99 (x2.3)	nd	Zheng et al., 2010
Rice glutelin B1 promoter	OsNAS1	Rice	Polished seeds	23.5 to 47 (x8.2)	5 (x1)	27.05 to 29.07 (x2.3)	nd	Zheng et al., 2010
Rice glutelin B1 promoter	OsNAS1	Rice	Leaves	60 ± 5 (x1)	18 ± 2 (1.8)	30 ± 2 (x1.9)	nd	Zheng et al., 2010
Average				186.3 (7.6)	45.0 (2.2)	46.4 (2.3)		

*Concentrations in μg/g field weight (FW) or μg/g dry weight (DW) are indicated with or without asterisks, respectively. Values inside parentheses represent the fold change in non-transgenic plants. nd, not determined; ref, reference.*

## Enhancement of NA Increased the Fe and Zn Concentrations in Plants

Nicotianamine plays an important role in metal transport in the plant body. It was suggested that NA is involved in the translocation of Fe and Zn into seeds in rice, *Arabidopsis*, tomato, and tobacco (Higuchi et al., 1996; Takahashi et al., 2003; Kim et al., 2005; Masuda et al., 2008,Masuda et al., 2009; Schuler et al., 2012). Fe is readily oxidized and precipitated in the apoplasm of both roots and shoots. Therefore, Fe uptake from the apoplasm is important for plant growth. In the *Arabidopsis* double *IRT1* and *Nramp1* mutant, Fe was precipitated and accumulated in the apoplast of the roots, while the Fe concentration in shoots was dramatically reduced compared with NT (Castaings et al., 2016). In *NAS*-overexpressing plants, the Fe and Zn concentrations were also increased (**Table 1**). NA might be involved in the mobilization of Fe and Zn in the apoplasm. In the seeds of *HvNAS1*-overexpressing soybean plants, the Fe and Zn concentrations increased to 110 and 65 µg/g DW, which were 2 and 1.45 times higher, respectively, than in NT plants (Nozoye et al., 2014a). In *HvNAS1*-overexpressing sweet potato, the Fe and Zn concentrations increased to 52.9 and 17 µg/g FW in the leaves and 15.1 and 3.5 µg/g DW in the storage roots, which were 3 and 3, and 2.1 and 3.5 times higher, respectively, than in NT plants (Nozoye et al., 2017). In *HvNAS1*-overexpressing tobacco, the Fe and Zn concentrations increased to 5.3 and 9.6 µg/g FW in the leaves, which were 5 and 2.3 times higher, respectively, than in NT plants (Kim et al., 2005). In *HvNAS1*-overexpressing rice via the CaMV 35S promoter, the Fe and Zn concentrations increased to 9 and 45 µg/g DW, respectively, in the polished seeds, which were 2 and 1.5 times higher than in NT seeds (Masuda et al., 2009). In contrast, in *HvNAS1*-overexpressing rice via the *OsActin1* promoter, the Fe and Zn concentrations were 170 and 25 µg/g DW in the leaves and 5 and 40 µg/g DW in the polished seeds, respectively, which did not differ significantly from those in NT plants (Masuda et al., 2009). By overexpressing rice *NAS* genes (*OsNAS1–3*), the Fe and Zn concentrations in rice seeds increased to 81 and 91 µg/g DW, respectively, which were 3.5 and 2.2 times higher than in NT seeds (Johnson et al., 2011). In *OsNAS1*-overexpressing rice via the maize ubiquitin promoter, the Fe and Zn concentrations in rice seeds were increased to 28 and 120 µg/g DW, respectively, which were 2.3 and 5.5 times higher than in NT seeds (Zheng et al., 2010). Seed-specific expression of *OsNAS1* under control of the rice glutelin promoter, increased the Zn concentration in rice polished seeds to 29.07 µg/g DW, which was 2.3 times higher than in NT seeds (Zheng et al., 2010); however, the Fe concentration was not altered in the polished seeds. In these plants, the Fe and Zn concentrations in leaves were increased to 18 and 30 µg/g DW, which were 1.8 and 1.9 times higher, respectively, than in NT leaves. As with the NA concentration, the Fe and Zn concentrations tended to be higher in soybean seeds; however, this difference was not significant compared to the NA concentration. In agreement, the increases in Fe and Zn concentrations were lower than those of the NA concentration (**Table 1**). The average increases in Fe and Zn were 2.2 and 2.3, respectively, whereas that of NA was 7.6. There might be a factor limiting the increases in the metals compared with NA. Because Fe and Zn are taken up from the soil via roots, modification of the uptake system might further increase Fe and Zn. It is also possible that the increased NA in NAS-overexpressing plants was not translocated in the plant body efficiently. There might be potential to increase Fe and Zn by changing the flow of NA in the plant body.

## *NAS*-Overexpressing Plants Showed Tolerance to Fe Deficiency

Several *NAS*-overexpressing plants have been confirmed to tolerate Fe deficiency compared to NT plants (Lee et al., 2009; Nozoye et al., 2014a,Nozoye et al., 2017). The plant growth is dramatically reduced under Fe-deficient conditions. The plant heights and soil and plant analyzer development (SPAD) values (which represent the chlorophyll content) of *NAS*-overexpressing soybean and sweet potato plants were higher than those of NT plants when grown in calcareous soil with low Fe availability, suggesting that these transgenic plants were conferred tolerance to Fe deficiency. Under normal soil conditions, their growth did not differ. In rice plants, the Fe-deficiency tolerance of *NAS*-overexpressing rice plants in calcareous soil was not determined. *HvNAS1-*overexpressing rice exhibits enhanced NAS activity in Fe-deficient roots (Higuchi et al., 2001) and contains a higher amount of NA and deoxymugineic acid than NT plants in both roots and shoots (Masuda et al., 2009). Transgenic rice lines expressing barley *NAS* genes exhibit increased tolerance to low Fe availability in calcareous soil (Suzuki et al., 2008). Rice plants overexpressing *OsIRO2*, a transcription factor that enhances expression of Fe deficiency-inducible genes including *OsNAS1* and *OsNAS2*, showed improved tolerance to low Fe availability in calcareous soil (Ogo et al., 2011). These results suggest that overexpression of the *NAS* gene in rice also enhances tolerance to Fe deficiency.

It was recently suggested that NA may be involved in Fe homeostasis; enhanced NA production induced Fe deficiency signaling and mobilization of Fe in the plant body (Nozoye et al., 2014b,Nozoye et al., 2014c). Since NA has the ability to chelate Fe, NA may enable the de-repression of Fe deficiency-inducible genes by drawing Fe from an unknown Fe-sensing mechanism, and further increase the NA and deoxymugineic acid (a primary MAs) levels. NA has long been considered a candidate long-distance Fe signaling molecule in both gramineous and dicot plants (Curie and Briat, 2003); however, this has not yet been proven. In rice, *NAS* overexpression positively modulates Fe homeostasis-related genes (Wang et al., 2013). NA accumulation in *Osnaat1* mutants triggers a constitutive Fe deficiency response (Cheng et al., 2007). In *Arabidopsis*, NA-over-accumulating plants showed an Fe-deficient phenotype and expressed Fe-inducible genes at higher levels than did NT plants; however, they also contained more Fe than did NT plants, suggesting that an increase in the NA apoplastic pool sequestered Fe, which controls plant Fe homeostasis (Cassin et al., 2009). The overexpression of *ZINC-INDUCED FACILITATOR 1* (*ZIF1*) in *Arabidopsis* increased the amount of NA in the roots and shoots and led to Fe deficiency (Haydon et al., 2012). ZIF1 is a vacuolar membrane-localized putative transporter required for Zn tolerance that is hypothesized to transport NA from the cytoplasm into the vacuoles. Perturbing the subcellular distribution of NA may have profound effects on Fe with respect to subcellular distribution and inter-organ partitioning. In agreement with this phenomenon, it was revealed that AtYSL1 and AtYSL3, Fe-NA transporters, are required for proper long-distance Fe signaling (Kumar et al., 2017). A *ysl1ysl3* double-mutant did not up- or down-regulate Fe deficiency-induced or -repressed genes, while it contained markedly low tissue levels of Fe compared to NT plants. These results suggest that NA may be involved in long-distance signaling to maintain Fe homeostasis. In *NAS*-overexpressing plants, the increased NA might induce Fe-deficiency-inducible genes that contribute to conferring tolerance to Fe deficiency.

## Conclusion

Overexpression of the *NAS* gene enhances NA levels in several crops, including crops in which endogenous NA is already high, such as soybean and sweet potato. Additionally, *NAS* overexpression enhances the Fe and Zn concentrations and confers tolerance to Fe deficiency in calcareous soil. The increase in NA tended to be higher in leaves than in seeds, while the increases in Fe and Zn were lower than those of NA. These results suggest the potential to increase NA, Fe, and Zn concentrations further in the edible parts of crops. Further analysis of NA translocation in the plant body will allow for improved engineering strategies not only to accumulate bioavailable Fe in edible parts, but also to increase the tolerance of plants to low Fe availability to meet the demands of plant production and to solve problems such as inadequate diet, food shortages, and global warming in the near future.

## Author Contributions

TN designed and wrote the manuscript.

## Conflict of Interest Statement

The author declares that the research was conducted in the absence of any commercial or financial relationships that could be construed as a potential conflict of interest.
